# Persistent Increase in Serum 25-Hydroxyvitamin D Concentration in a Dog Following Cholecalciferol Intoxication

**DOI:** 10.3389/fvets.2019.00472

**Published:** 2020-01-09

**Authors:** Charlotte Gerhard, Jared A. Jaffey

**Affiliations:** Department of Small Animal Medicine and Surgery, College of Veterinary Medicine, Midwestern University, Glendale, AZ, United States

**Keywords:** rodenticide, hypercalcemia, vitamin D, 25(OH)D, 25-hydroxyvitamin D

## Abstract

Cholecalciferol is becoming an increasingly utilized rodenticide in the United States due to changes implemented by the Environmental Protection Agency (EPA) to reduce unintended exposure of wildlife to second-generation anticoagulant rodenticides. The lipophilic properties of cholecalciferol and prolonged tissue elimination are well-documented; however, long-term clinical ramifications are unknown. This report describes unique clinicopathologic and treatment features during the acute phase of cholecalciferol rodenticide toxicosis in a 4-year-old neutered Shih-Tzu mix that presented for intermittent vomiting, anorexia, polyuria, and polydipsia. In addition, this report also highlights the potential benefit of serial measurements of serum 25-hydroxyvitamin D concentrations and long-term treatment in the chronic phase of cholecalciferol rodenticide toxicosis in dogs.

## Background

Cholecalciferol is an increasingly utilized rodenticide in the United States. This shift in rodenticide usage was influenced by changes in Environmental Protection Agency (EPA) regulations that removed most second-generation anticoagulant rodenticides from the over-the-counter market (1, 2). In accordance with this new regulation, d-CON (d-CON, Reckitt Benckiser LLC, Slough, England, United Kingdom), the largest manufacture of rodent control products, switched to cholecalciferol based rodent baits in early 2015. The high lipid solubility of cholecalciferol and its metabolites prolongs tissue elimination. The long-term clinical ramifications of the persistent release of stored cholecalciferol from tissues in dogs that inadvertently ingest toxic amounts of this rodenticide is unknown. This report describes for the first time the clinical presentation, clinicopathologic abnormalities, and treatment of a dog in the acute and chronic phases of cholecalciferol rodenticide toxicosis.

## Case Presentation

**Figure 1:**
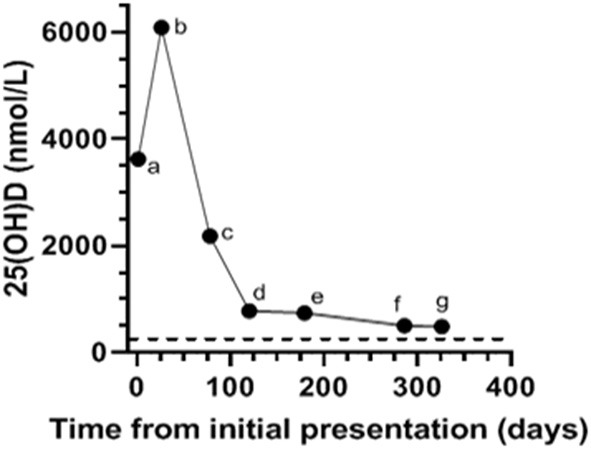
Serial serum 25-hydroxyvitamin (OH)-D concentrations in a dog with cholecalciferol rodenticide toxicosis starting at the time of initial presentation (a; Day 1) as well as on subsequent evaluations on (b; Day 26, c; Day 78, d; Day 120, e; Day 179, f; Day 286, g; Day 326). The dog received prednisone (0.6 mg/kg PO q24 h), aluminum hydroxide (16 mg/kg PO q12 h), and Hills Prescription Diet k/d (Hill's Pet Nutrition Inc., Topeka, KS) from the time of hospital discharge (day 8) to (f; Day 286). Serum 25(OH)D measured at Michigan State University (MSU) Diagnostic Center for Population and Animal Health with a commercially available and validated radioimmunoassay. The reference interval for canine serum 25(OH)D established by MSU is 109–423 nmol/L. The dotted black line denotes the upper limit of the reference interval.

A 4-year-old neutered Shih Tzu mix weighing 8.0 kg (17.6 lb), presented to Midwestern University College of Veterinary Medicine with a 72 h history of intermittent vomiting, anorexia, lethargy, polyuria, and polydipsia (day of initial examination designated as day 1). The dog was reported by the owner to have ingested an unknown amount of cholecalciferol rodenticide (TERAD_3_ Ag BLOX, Bell Laboratories Inc., Madison, WI). The timing of ingestion was not initially known but was subsequently determined to have occurred ~4–6 days before presentation. Pertinent physical examination findings included a heart rate of 140 beats per minute, respiratory rate of 30 breaths per minute, body condition score of 7/9, and a rectal temperature of 100.7°F (38.2°C). Serum biochemical parameters outside the reference interval were total calcium (>16 mg/dL; reference interval 8.6–11.8 mg/dL), creatinine (1.6 mg/dL; reference interval 0.3–1.4 mg/dL), and glucose (121 mg/dL; reference interval 60–110 mg/dL), and ionized calcium (1.93 mmol/L; reference interval 1.20–1.32 mmol/L). The serum phosphorus was within the reference interval (4.4 mg/dL; reference interval 2.9–6.6 mg/dL; Table 1). A urinalysis revealed a urine specific gravity of 1.008. The complete blood count did not reveal any abnormalities. Serum 25-hydroxy (OH)D concentration on day 1 (~3 days after cholecalciferol rodenticide ingestion) was markedly increased (3,625 nmol/L; reference interval 109–423 nmol/L; Figure 1). Serum 25(OH)D concentration was not initially evaluated because of the known history of cholecalciferol rodenticide ingestion. Instead, serum 25(OH)D concentration was measured retrospectively with serum that had been obtained on day 1 and stored for 9 months in a freezer resistant plastic tube at −20°C.

**Table 1:** Serial serum biochemical parameters, ionized calcium concentration, and hematocrit in a dog with cholecalciferol rodenticide toxicosis starting at the time of initial presentation (day 1) until the day of discharge (day 8).

	**Day 1**	**Day2** **(10:23)**	**Day2** ** (16:48)**	**Day 3**	**Day 4**	**Day 5**	**Day 7**	**Reference values^a^^,^^b^**
Calcium	>16	–	–	–	13	11.2	–	8.8–11.8 mg/dL
Phosphorous	4.4	–	–	–	5.4	2.9	–	2.9–6.6 mg/dL
Creatinine	1.6	–	1.5	1.2	1.1	1.3	0.89	0.3–1.4 mg/dL
Blood urea nitrogen	22	–	14.0	13.0	14.0	26.0	–	7–25 mg/dL
Sodium	141	135	146	150	144	141	144	139–150 mmol/L
Potassium	4.0	2.9	3.0	3.9	4.1	3.4	4.5	3.4–4.9 mmol/L
Hematocrit	52	55	56	47	40	50	43	35–50%
Ionized calcium	1.93	1.57	1.23	1.41	1.60	1.07	1.26	1.12–1.40 mmol/L

a *Ionized calcium and hematocrit measured with i-STAT 1 Handheld Analyzer, Abaxis Inc., Union City, CA*.

b *Biochemical parameters measured with VETSCAN VS2 Chemistry Analyzer, Abaxis Inc., Union City, CA*.

Upon hospitalization initial therapy overnight consisted of IV sodium chloride 0.9% with the rate calculated to support maintenance requirements and promote calciuresis (180 ml/kg/day). Additional therapeutic management the evening of presentation consisted of activated charcoal without sorbitol (2.5 ml/kg PO q6 h), and intralipids (1.5 ml/kg IV bolus, followed by 0.25 ml/kg/min IV over 1 h). The following morning (day 2), the following treatments were instituted furosemide [2 mg/kg IV bolus, followed by 1 mg/kg/h constant rate of infusion (CRI)], dexamethasone (0.1 mg/kg/day IV), and aluminum hydroxide (10 mg/kg PO q8 h). Following ~8 h of this therapy (day 2), the dog became dehydrated. The dog was tachycardic (heart rate of 170 beats per minute), developed tacky mucous membranes and a prolonged skin turgor as well as a decrease of 0.6 kg or 7.5% of body weight. A blood-gas analysis revealed static azotemia (creatinine 1.5 mg/dL) and an ionized calcium within the reference interval (1.23 mmol/L). Supportive care was continued including 0.9% sodium chloride supplemented with 25 mEq potassium chloride (KCl)/L with the fluid rate adjusted to 270 ml/kg/day. The furosemide CRI was discontinued due to normalization of the ionized calcium within the reference interval as well as the development of moderate dehydration. Prednisone (1.0 mg/kg PO q24 h) was substituted for dexamethasone and other medications including maropitant (1.0 mg/kg IV q24 h), and ondansetron (0.5 mg/kg PO q12 h) were initiated.

On day 4, the ionized hypercalcemia had returned (1.6 mmol/L) along with a mild total hypercalcemia (13.0 mg/dL; Table 1). Sodium chloride 0.9% with 22 mEq KCl/L was administered with the rate adjusted to 180 ml/kg/day and furosemide (0.5 mg/kg/h CRI) was reinstituted in addition to zoledronate (0.3 mg/kg, diluted in 100 ml 0.9% sodium chloride administered IV over 2 h), because of the worsening ionized hypercalcemia. The next day (day 5) ionized hypocalcemia was identified (1.07 mmol/L) along with a stable creatinine (1.3 mg/dl), but increased blood urea nitrogen (26 mg/dl; reference interval 7–25 mg/dl). The furosemide CRI was discontinued due to resolution of ionized hypercalcemia and sodium chloride 0.9% containing 15 mEq KCl/L was continued with an adjusted rate to 90 ml/kg/h. The dog was discharged on day 8 with resolution of azotemia and ionized hypercalcemia. Medical management at the time of discharge included prednisone (0.6 mg/kg PO q24 h), aluminum hydroxide (16 mg/kg PO q12 h), and Hills Prescription Diet k/d (Hill's Pet Nutrition Inc., Topeka, KS).

The dog was presented for a recheck on day 12. The owner reported that the dog remained bright, alert, interactive, and maintained a good appetite from the time of discharge (day 8) to this first recheck examination. A serum biochemical profile was performed and revealed total hypercalcemia (11.8 mg/dL), as well as phosphorus (4.3 mg/dL), creatinine (1.0 mg/dL), and blood urea nitrogen (15 mg/dL) within their respective reference intervals. The ionized calcium (1.37 mmol/L) was at the upper end of the reference interval (Table 2). Therapy with prednisone, aluminum hydroxide, and Hills Prescription Diet k/d food remained unchanged because of the persistent total hypercalcemia. The dog remained clinically unchanged and was evaluated again on day 26. A recheck examination was requested for 1 week (day 19), but the owner did not comply with that recommendation. At this visit pertinent serum biochemical parameters and ionized calcium concentration were within reference intervals (Table 2). Serum 25(OH)D concentration was evaluated to facilitate the decision of whether to continue the current course of therapy. The discontinuation of therapy with persistence of severe hypervitaminosis D, theoretically, could have resulted in recrudescence of hypercalcemia and organ damage. The serum 25(OH)D concentration was still markedly increased (6,091 nmol/L; Figure 1). The treatment regimen remained unchanged because of the continued severe hypervitaminosis D. The dog was subsequently evaluated approximately once every 4–8 weeks. The dog continued treatment with prednisone and aluminum hydroxide at the previous dosages and was fed Hills Prescription Diet k/d food until day 286. At that time the dog was transitioned to a commercial maintenance dog food and aluminum hydroxide as well as prednisone were discontinued. While all parameters on serum biochemical profiles and ionized calcium concentrations remained within their respective reference intervals (Table 2), the serum 25(OH)D concentration, remained mildly above the reference interval, day 326 (Figure 1).

**Table 2:** Serial serum biochemical parameters, ionized calcium concentration, and hematocrit in a dog with cholecalciferol rodenticide toxicosis during the chronic phase starting at the first recheck after hospital discharge (day 12) until the day 326.

	**Day 12**	**Day 26**	**Day 78**	**Day 120**	**Day 179**	**Day 244**	**Day 286**	**Day 326**	**Reference values^a^^,^^b^**
Calcium	11.8	9.5	10.7	10.3	10.0	9.8	10.8	10.3	8.6–11.8 mg/dL
Phosphorous	4.3	2.9	4.1	3.2	3.7	3.7	4.4	3.3	2.9–6.6 mg/dL
Creatinine	1.0	0.8	0.6	0.6	0.8	0.8	1.2	1.1	0.3–1.4 mg/dL
Blood urea nitrogen	15.0	9.0	7.0	5.0	8.0	6.0	19.0	14	6–31 mg/dL
Sodium	147	144	148	143	146	145	148	147	139–154 mmol/L
Potassium	4.7	4.3	4.3	4.4	4.4	4.5	4.5	4.3	3.6–5.5 mml/L
Hematocrit	44	50	40	39	38	40	–	43	35–50%
Ionized calcium	1.37	1.07	1.32	1.26	1.13	1.32	–	1.29	1.12–1.40 mmol/L

a *Ionized calcium and hematocrit measured with i-STAT 1 Handheld Analyzer, Abaxis Inc., Union City, CA*.

b *Biochemical parameters measured with AU680 Clinical Chemistry Analyzer, Beckman Coulter Inc., Atlanta, GA*.

## Discussion

The current shift of rodenticide usage from second-generation anticoagulant rodenticides to over-the-counter compounds is reflective of EPA regulations implemented to reduce the risk of exposure to wildlife (3, 4). Although an important step in protecting wildlife, the increased availability of vitamin D based rodent baits have resulted in new and challenging problems in companion animals. Rodenticide ingestion was the seventh most commonly reported toxin to the ASPCA Animal Poison Control Center accounting for 13,468 cases in 2018 (5). The current report describes unique clinicopathologic and treatment features during the acute phase of cholecalciferol toxicosis. Moreover, it also highlights the potential benefit of serial measurements of serum 25(OH)D concentrations and long-term treatment in the chronic phase of cholecalciferol rodenticide toxicosis in dogs.

Cholecalciferol toxicosis in dogs is typically characterized by hypercalcemia, hyperphosphatemia, and azotemia (6). Hyperphosphatemia, hypercalcemia (total and ionized), and azotemia typically develop within 12, 24, and 72 h, respectively, after ingestion of toxic amounts of cholecalciferol (7, 8). Interestingly, hyperphosphatemia was never identified in this dog despite the development of both hypercalcemia (total and ionized) and mild azotemia. The specific reason for the absence of hyperphosphatemia in this dog is unknown and is likely multifactorial. One potential explanation is that this dog had a rapid and robust increase in fibroblast growth factor (FGF)23, a key hormone in the phosphatonin system, which is integral to phosphorus metabolism. This could have efficiently mitigated excessive serum phosphate accumulation. This phosphate wasting hormone is upregulated by calcitriol (the biologically active form of vitamin D) as well as phosphorus and causes renal phosphate wasting, reduces intestinal phosphate absorption, and decreases activation of calcitriol (9). Studies investigating FGF23 in dogs are limited to its role in chronic kidney disease (10). However, a recent clinical trial identified a significant increase in plasma FGF23 concentration following oral administration of high doses of cholecalciferol to healthy humans (11). While the mechanisms by which this dog maintained serum phosphorus concentrations within the reference interval can be speculated, this report highlights that hyperphosphatemia is not uniformly identified in dogs with cholecalciferol toxicity. In other words, vitamin D toxicity should not be ruled out in dogs with an acute onset of polyuria, polydipsia, lethargy, vomiting anorexia, and hypercalcemia when the serum or plasma phosphorus concentration is within the reference interval. The measurement of serum 25(OH)D can provide conclusive evidence of vitamin D toxicosis.

Treatment of cholecalciferol rodenticide toxicity in dogs that present to a veterinarian in the acute phase revolves around emesis and administration of an adsorbent if ingestion is known to have occurred within ~4–8 h before presentation. Beyond this peracute presentation, therapy is aimed at supportive care for hypercalcemia and any pathologic sequela of this electrolyte disturbance (e.g., acute kidney failure, seizures, arrhythmias, vomiting, and diarrhea) (5). Adsorbents including activated charcoal, cholestyramine, and less commonly colestipol are used to bind ingested toxic compounds to prevent absorption and enterohepatic recirculation. Cholecalciferol has extensive enterohepatic recirculation and thus adsorbents could be beneficial (12). This form of decontamination is optimal when pursued within 24 h of toxin ingestion (12). Initially, the owner was unaware of when the rodenticide ingestion occurred. Activated charcoal was administered to the dog because of the initial uncertainty surrounding the timing of rodenticide ingestion. Cholestyeramine, an anion exchange resin binds with lipoproteins and bile acids, thereby inhibiting intestinal absorption of toxic substances as well as preventing enterohepatic recirculation (13). This adsorbent could have potentially been administered in addition to activated charcoal owing to its superior ability to bind highly lipophilic compounds like cholecalciferol (12, 13). This product was not available the evening of initial presentation.

Hypercalcemia is one of the hallmark clinicopathologic features of cholecalciferol rodenticide toxicity in companion animals and can result in cell death as well as tissue mineralization in the central nervous system, heart, gastrointestinal tract, and kidneys (1, 7, 14). Therefore, strategies to decrease ionized hypercalcemia via promotion of calciuresis and inhibition of calcium mobilization from bone are integral. Calciuresis can be enhanced via administration of 0.9% sodium chloride fluids, furosemide, and glucocorticoids (7). Physiologic saline promotes calciuresis through increased glomerular filtration rate as well as reduced renal tubular calcium reabsorption that occurs secondary to increased filtered sodium (7). Administration of furosemide reduces calcium reabsorption in the loop of Henle. Interestingly, administration of furosemide in people with hypercalcemia is controversial and many have discontinued its use in these cases because a lack of evidence-based data and potential risk for excessive diuresis and dehydration (15, 16). It is important to maintain hydration at all times. Dehydration will decrease delivery of furosemide to the proximal tubules where its effects in the ascending loop of Henle are exerted. Moreover, dehydration can potentiate hypercalcemia and kidney injury from contraction of extracellular fluid volume (17). The dog in this report became dehydrated within ~8 h of initiating therapy with furosemide. This could have been related to the aggressive dose of furosemide used in this dog. The recommended dosing instructions for furosemide administration in dogs with vitamin D toxicosis includes an initial intravenous loading dose of 0.66–1.0 mg/kg followed by a CRI of 0.66–1.0 mg/kg/h (7, 12). Glucocorticoids prevent renal reabsorption of calcium and thus promotes excretion (7).

Intravenous lipid emulsion (ILE) is an alternative potential therapy that could mitigate the development of hypercalcemia secondary to hypervitaminosis D. These fat emulsions can form chylomicron droplets in serum, which act as a “sink” for highly lipophilic compounds like vitamin D (13). The binding of vitamin D in this “sink” would theoretically make this compound unavailable to bind to vitamin D receptor, the only receptor capable of translating vitamin D mediated actions. In addition, the increased serum lipid concentration is thought to form a concentration gradient between tissue and serum, causing lipophilic drugs (i.e., vitamin D) to move away from deposits in tissue (18). A recent case report highlighted the potential of ILE to decrease serum 25(OH)D concentration following vitamin D toxicosis in a dog (18). It is unknown if ILE administration to the dog in this report mitigated the magnitude of hypercalcemia. Future studies focusing on the benefit of ILE in dogs after vitamin D toxicosis are needed.

Dogs that are severely affected or whose ionized hypercalcemia persist despite therapy require more intensive treatment. Bisphosphonates are the preferred class of drug in these cases because they often decrease the calcium concentration within 24–48 h after administration through inhibition of osteoclast mediated bone resorption (19). Historically, pamidronate has been the bisphosphonate of choice in dogs with severe or refractory hypercalcemia secondary to cholecalciferol rodenticide toxicity (20, 21). However, zoledronate is a more potent bisphosphonate and considered the first line option in humans with hypercalcemia (22). The time to normocalcemia following zoledronate administration in people is variable. One clinical trial that evaluated its use in people with cancer found that normocalcemia occurred 2–3 days after administration and lasted for more than 21 days (23). Overall, there is a paucity of information regarding the use of zoledronate in dogs. There is a single report in four pet dogs with hypercalcemia of malignancy (22). The time to normocalcemia in that report varied among the three dogs in which it was achieved. Recrudescence of hypercalcemia was appreciated 2 weeks after zoledronate was administered to the two dogs for which follow up data was available. To the authors' knowledge, this is the first report describing the use of zoledronate in a dog with hypercalcemia secondary to cholecalciferol rodenticide toxicosis. Similar to the findings in the aforementioned case series, our report reinforces that zoledronate is a viable alternative to pamidronate in dogs with hypercalcemia secondary to cholecalciferol toxicosis. Studies in dogs are needed to investigate the details of zoledronate administration (i.e., dose, time to and duration of normocalemia) among the various disease etiologies causing hypercalcemia.

The high lipid solubility of cholecalciferol and resulting metabolites contribute to its prolonged tissue elimination resulting in persistent marked hypercalcemia (12). After absorption, cholecalciferol is either converted within the liver to 25(OH)D, the major circulating form of vitamin D, or stored within adipose tissue (2). Importantly, the storage of cholecalciferol in adipose tissue serves as a reservoir that continuously leaches into systemic circulation. The conversion of cholecalciferol to 25(OH)D is dependent on substrate availability with limited negative feedback (19). Typically, the activation of 25(OH)D in the kidney into calcitriol is efficiently inhibited in the presence of hypercalemia. However, even this tightly regulated negative feedback system can be circumvented by markedly increased 25(OH)D concentrations, which can activate vitamin D receptors as well as displace calcitriol from its carrier protein resulting in excessive “free” calcitriol concentrations (24–26). The reported half-life of serum 25(OH)D following cholecalciferol rodenticide toxicosis is variable and likely longer than reported in the veterinary literature, as demonstrated by a recent human study that examined vitamin D storage following administration of oral cholecalciferol for 5 years (27). Interestingly, that study identified that the serum half-life of 25(OH)D and thus rate of elimination varied with time. There was a negative trend in serum 25(OH)D concentrations that was fastest in the first 3 months and yielded a half-life of 83 days. The half-life of serum 25(OH)D thereafter was 255 days, indicating a much slower decrease of 25(OH)D after the first 3 months (27). A similar pattern of elimination was demonstrated in our dog where serum 25(OH)D concentrations substantially decreased in the first 4 months and then proceeded to gradually decrease thereafter. This parallel suggests the possibility that the clinical consequences of cholecalciferol rodenticide toxicosis in dogs extend beyond the acute phase and long-term treatment could be warranted in some dogs to prevent or reduce the harmful sequela of this toxicosis.

Serum 25(OH)D concentrations remained greater than the upper limit of the reference interval for almost a year after the dog in this report ingested cholecalciferol rodenticide. The persistence of hypervitaminosis D combined with the initial azotemia prompted the recommendation for long-term therapy with prednisone, aluminum hydroxide, and Hills Prescription Diet k/d food. Hypercalcemia can be seen in humans with serum 25(OH)D concentrations >500 nmol/L (26). The dog in this report maintained serum 25(OH)D concentrations >500 nmol/L for 9 months following cholecalciferol rodenticide ingestion, which suggests there was a potential for long-term consequences (e.g., tissue mineralization in the central nervous system, heart, gastrointestinal tract, and kidneys) associated with this toxicity had long-term therapy not been instituted. An early study in 1947 that investigated the effects of a single massive dose of oral vitamin D (300,000–530,000 IU/kg of vitamin D_2_) in dogs found that hypercalcemia persisted for 6 months in all dogs that survived the acute phase of toxicity. The only treatment these dogs received after demonstration of clinical signs was a single oral dose of 1,000,000 IU vitamin A. Extensive calcification was identified in the lungs, hearts, and kidneys of all dogs in that study (14). Future studies are needed to investigate the long-term benefit of treatment beyond the acute phase of cholecalciferol rodenticide toxicity. However, serial serum 25(OH)D measurements could provide the clinician support with therapeutic decision making on a case by case basis. The turn-around time for serum 25(OH)D measurement varies with commercial laboratory used but commonly takes 1–3 days.

In summary, vitamin D toxicosis presents a clinical challenge in that the current recommended treatment interval could be insufficient. The lipophilic properties and long half-life suggest that prolonged treatment expanding beyond weeks or even months could be necessary to mitigate soft tissue mineralization and possible organ damage. The unique clinicopathologic features in this case highlight that hyperphosphatemia is not uniformly identified in all dogs with vitamin D toxicosis and should not be ruled out especially in cases presenting with limited history. Serial measurement of 25(OH)D can provide the clinician with conclusive evidence of vitamin D toxicosis and assist with clinical decisions in the acute and chronic phases.

## Data Availability Statement

All datasets generated for this study are included in the article.

## Author Contributions

JJ and CG contributed to the writing of the manuscript, literature review, and the final review of the manuscript.

### Conflict of Interest

The authors declare that the research was conducted in the absence of any commercial or financial relationships that could be construed as a potential conflict of interest.

## References

[1] Mackenzie CP Burnie AG Heads KW Poisoning in four dogs by a compound containing warfarin and calciferol. J Small Anim Pract. (1987) 28:433–45. 10.1111/j.1748-5827.1987.tb01437.x

[2] DeClementi C Sobczak BR. Common rodenticide toxicoses in small animals. Vet Clin N Am Small Anim Pract. (2018) 48:1027–38. 10.1016/j.cvsm.2018.06.006 30173927

[3] Elliott JE Rattner BA Shore RF Brink NW Paying the pipers: mitigating the impact of anticoagulant rodenticides on predators and scavengers. Bioscience. (2016) 66:401–7. 10.1093/biosci/biw028

[4] Environmental Protection Agency Risk Mitigation Decision for Ten Rodenticides. (2008). Retrieved from: https://www.regulations.gov/document?D=EPA-HQ-OPP-2006-0955-0764 (accessed June 24, 2019).

[5] Just In: Announcing the Top 10 Toxins of 2018. ASCPA (2019). Retrieved from: https://www.aspca.org/news/juts-announcing-top-10-toxins-2019 (accessed August 9, 2019).

[6] Rumbeiha W Braselton W Nachreiner R Refsal K. The postmortem diagnosis of cholecalciferol toxicosis: a novel approach and differentiation from ethylene glycol toxicosis. J Vet Diagn Invest. (2000) 12:426–32. 10.1177/104063870001200506 11021429

[7] DiBartola SP Disorders of Calcium: Hypercalcemia and Hypocalcemia. Fluid, Electrolyte, and Acid-Base Disorders in Small Animal Practice. 4th ed. St. Louis, MO: Saunders/Elsevier (2011). p. 121–87.

[8] Poppenga RH Gwaltney-Brant S Small Animal Toxicology Essentials. Chichester: Wiley-Blackwell (2011). p. 121–2.

[9] Hardcastle MR Dittmer KE. Fibroblast growth factor 23: a new dimension to diseases of calcium-phosphorus metabolism. Vet Pathol. (2015) 52:770–84. 10.1177/0300985815586222 26018436

[10] Dittmer KE Perera KC Elder PA. Serum fibroblast growth factor 23 concentrations in dogs with chronic kidney disease. Res Vet Sci. (2017) 114:348–50. 10.1016/j.rvsc.2017.06.013 28667925

[11] Nygaard B Frandsen NE Brandi L Rasmussen K Oestergaard OV Oedum L. Effects of high doses of cholecalciferol in normal subjects: a randomized double-blinded, placebo-controlled trial. PLoS ONE. (2014) 9:e102965. 10.1371/journal.pone.0102965 25166750 PMC4148309

[12] Peterson M Fluegeman K. Cholecalciferol. Top Companion Anim Med. (2013) 28:24–7. 10.1053/j.tcam.2013.03.006 23796485

[13] Peterson ME Toxicologic Decontamination. Small Animal Toxicology. 3rd ed. St. Louis, MO: Saunders/Elsevier (2013). p. 73–83.

[14] Morgana F Axelrodh E Groody M The effect of a single massive dose of vitamin D on young dogs. Am J Physiol. (1947) 149:333–9. 10.1152/ajplegacy.1947.149.2.333 20239961

[15] Maier JD Levine SN. Hypercalcemia in the intensive care unit: a review of pathophysiology, diagnosis, and modern therapy. J Intensive Care Med. (2015) 30:235–52. 10.1177/0885066613507530 24130250

[16] Carrick AI Costner HB. Rapid fire: hypercalcemia. Emerg Med Clin N Am. (2018) 36:549–55. 10.1016/j.emc.2018.04.008 30037441

[17] Kruger JM Osborne CA Nachreiner RF Refsal KR. Hypercalcemia and renal failure. Vet Clin N Am Small Anim Pract. (1996) 26:1417–45. 10.1016/S0195-5616(96)50135-X 8911026

[18] Perry BH McMichael M Rick M Jewell E. Reduction of serum 25-hydroxyvitamin D concentrations with intravenous lipid emulsion in a dog. Can Vet J. (2016) 57:1284–6. 27928177 PMC5109633

[19] Morrow C Cholecalciferol poisoning. Vet Med. (2001) 96:905–11.

[20] Hostutler RA Chew DJ Jaeger JQ Klein S Henderson D DiBartola SP. Uses and effectiveness of pamidronate disodium for treatment of dogs and cats with hypercalcemia. J Vet Intern Med. (2005) 19:29–33. 10.1111/j.1939-1676.2005.tb02654.x 15715044

[21] Rumbeiha WK Fitzgerald SD Kruger JM Braselton WE Nachreiner R Kaneene JB. Use of pamidronate disodium to reduce cholecalciferol-induced toxicosis in dogs. Am J Vet Res. (2000) 61:9–13. 10.2460/ajvr.2000.61.9 10630770

[22] Schenk A Lux C Lane J Martin O. Evaluation of zoledronate as treatment for hypercalcemia in four dogs. J Am Anim Hosp Assoc. (2018) 54:e54604. 10.5326/JAAHA-MS-6681 30272485

[23] Body JJ Lortholary A Romieu G Vigneron AM Ford J. A dose-finding study of zoledronate in hypercalcemic cancer patients. J Bone Miner Res. (1999) 14:1557–61. 10.1359/jbmr.1999.14.9.1557 10469284

[24] Lou Y Laaksi I Syvälä H Bläuer M Tammela TLJ Ylikomi T. 25-hydroxyvitamin D3 is an active hormone in human primary prostatic stromal cells. FASEB J. (2004) 18:332–4. 10.1096/fj.03-0140fje 14657005

[25] Pettifor JM Bikle DD Cavaleros M Zachen D Kamdar MC Ross FP. Serum levels of free 1,25-dihydroxyvitamin D in vitamin D toxicity. Ann of Intern Med. (1995) 122:511. 10.7326/0003-4819-122-7-199504010-00006 7872586

[26] Vieth R. The mechanisms of vitamin D toxicity. Bone Miner. (1990) 11:267–72. 10.1016/0169-6009(90)90023-9 2085680

[27] Martinaityte I Kamycheva E Didriksen A Jakobsen J Jorde R. Vitamin D stored in fat tissue during a 5-year intervention affects serum 25-hydroxyvitamin D levels the following year. J Clin Endocrinol Metab. (2017) 102:3731–8. 10.1210/jc.2017-01187 28973683

